# Assessment of Sub-Micron Particles by Exploiting Charge Differences with Dielectrophoresis

**DOI:** 10.3390/mi8080239

**Published:** 2017-08-02

**Authors:** Maria F. Romero-Creel, Eric Goodrich, Danielle V. Polniak, Blanca H. Lapizco-Encinas

**Affiliations:** Microscale Bioseparations Laboratory and Biomedical Engineering Department, Rochester Institute of Technology, Rochester, NY 14623, USA; mfr2129@rit.edu (M.F.R.-C.); emg1035@rit.edu (E.G.); dp7914@mail.rit.edu (D.V.P.)

**Keywords:** dielectrophoresis, electrical double layer, electrokinetics, particle polarization

## Abstract

The analysis, separation, and enrichment of submicron particles are critical steps in many applications, ranging from bio-sensing to disease diagnostics. Microfluidic electrokinetic techniques, such as dielectrophoresis (DEP) have proved to be excellent platforms for assessment of submicron particles. DEP is the motion of polarizable particles under the presence of a non-uniform electric field. In this work, the polarization and dielectrophoretic behavior of polystyrene particles with diameters ranging for 100 nm to 1 μm were studied employing microchannels for insulator based DEP (iDEP) and low frequency (<1000 Hz) AC and DC electric potentials. In particular, the effects of particle surface charge, in terms of magnitude and type of functionalization, were examined. It was found that the magnitude of particle surface charge has a significant impact on the polarization and dielectrophoretic response of the particles, allowing for successful particle assessment. Traditionally, charge differences are exploited employing electrophoretic techniques and particle separation is achieved by differential migration. The present study demonstrates that differences in the particle’s surface charge can also be exploited by means of iDEP; and that distinct types of nanoparticles can be identified by their polarization and dielectrophoretic behavior. These findings open the possibility for iDEP to be employed as a technique for the analysis of submicron biological particles, where subtle differences in surface charge could allow for rapid particle identification and separation.

## 1. Introduction

The separation and assessment of submicron particles are essential processes in chemical and biological analysis, with particular importance in the fields of nanotechnology and biotechnology. Microfluidic devices, due to their small size, are an ideal platform for the examination of submicron particles. Electrokinetic (EK) methods have proven to be one of the leading microfluidic techniques for the assessment, separation, and enrichment of nano- to micron-sized particles. Electric field driven techniques offer great flexibility, since a single stimulation force can be used to move both the particles and the suspending medium. Electroosmotic flow (EOF) is commonly used as a liquid and particle pumping mechanism due to the attractive advantage of requiring no mechanical parts, as this phenomenon exploits the electrical double layer (EDL) of the device substrate material [1]. However, EOF requires high voltages, which can lead to undesirable effects such as electrolysis and Joule heating [2,3,4]. Electrophoresis (EP) is another important EK mechanism that refers to the motion of charged particles relative to the suspending medium, i.e., EP exploits particles’ charge differences to enable particle separations; where distinct particles exhibit differential migration under the influence of an electric field. EP is a well-known phenomenon, used commonly as gel EP for the separation of proteins and DNA in many applications. Other variations of EP, such as capillary EP, isoelectric focusing, isotachophoresis, electrochromatography, and micellar electrokinetic chromatography, are also commonly used successful techniques for separating nano and micron-sized particles by exploiting electrical charge differences [5,6]. 

Dielectrophoresis (DEP) is a peculiar electric-field driven technique since it exploits particle polarization effects, not electrical charge, when particles are exposed to a non-uniform electric field [1]. Particles acquire a dipole moment under the effect of an electric field, the poles in this dipole moment are subjected to several forces which results in a net dielectrophoretic force acting on the particle [7]. This method has received significant attention [8,9,10,11,12,13,14,15] due to its flexibility, as DEP can occur in AC and DC electric fields and it can be used to manipulate charged and non-charged particles. There is a plethora of reports available in the literature focused on the dielectrophoretic analysis of macromolecules, viruses, and other submicron particles [16,17,18,19,20,21]. 

DEP offers great potential for the separation and enrichment of particles by exploiting slight differences in particle polarization—which is dictated by particle characteristics such as shape, size, and dielectric properties. Depending on the sign of the dipole moment, particles move in distinct directions [7] and can exhibit positive or negative dielectrophoretic behavior. A particle that is more polarizable than the suspending medium will migrate towards the regions of higher electric field gradient under positive DEP; while particles with lower polarizability than the medium would migrate away from these regions due to negative DEP [22]. Particle polarization is a complex phenomenon; many groups have studied the polarization of submicron particles and its relation to particle dielectrophoretic behavior [7,21,22,23,24,25,26,27,28,29,30]. Differences in dielectric properties, such as surface charge, can be exploited to achieve effective particle separation. Green and Morgan [25] separated 93 nm diameter particles into two subpopulations by means of DEP with AC potentials at high frequencies (MHz range). In later studies [26,27], this group characterized the dielectrophoretic response of sub-micron particles as a function of electrolyte composition and conductivity, frequency of the applied potentials and particle size. In particular, they analyzed the effects of interfacial polarization mechanism and EDL polarization. The effect of surface functionalization has also been studied [28] demonstrating that a reduction on particle surface conductance leads to changes in the dielectrophoretic behavior of the particles. 

The DEP response of submicron particles under the influence of high frequency (>1000 Hz) electric potential is well documented in the literature by excellent studies [25,26,27,28,29,30]. However, the behavior of these particles at low frequencies (<1000 Hz) is still an unexplored area. Insulator-based DEP (iDEP), a dielectrophoretic mode where insulating structures are employed to create non-uniform electric fields [31,32]; usually employs low frequency AC and DC electric potentials to achieve particle manipulation [15,23,33,34,35,36]. In iDEP microsystems, particles are exposed to several forces simultaneously (EOF, EP, and DEP), and the resulting particle motion is the net migration caused by the combination of all present forces [37,38]. Particles can be “trapped” between the insulating structures if the applied electric field is high enough to produce DEP forces than can overcome all other present forces [31]; and particles will “stream” along the electric field lines when the generated DEP forces are just comparable to EOF and EP [32]. The interplay between these forces can be fine-tuned to achieve a desired dielectrophoretic process, from particle identification to the enrichment of low-abundant species [39]. 

The present work is focused on studying how differences in a particle’s electrical charge can be used to achieve dielectrophoretic separation of particles or detect changes in a particle’s surface composition, employing DC and low frequency (<1000 Hz) electric potentials. This low frequency range has not been fully explored in dielectrophoretic microsystems. This work is organized as follows: We first discuss the fundamentals of dielectrophoretic force and particle polarization and the acquisition of a dipole moment. We follow this discussion with mathematical predictions obtained with COMSOL Multiphysics^®^ (Version 4.4, COMSOL Inc., Burlington, MA, USA) of the motion of submicron polystyrene particles under positive and negative DEP. Our experimental results demonstrate that is possible to identify and separate submicron particles with similar characteristics (same size, same shape, and same substrate material) by means of iDEP by exploiting differences in surface charge. These findings open the possibility for iDEP to be employed as a technique for the analysis of submicron biological particles, where subtle differences in surface charge could allow for rapid particle identification and separation. 

## 2. Theory

### 2.1. Dielectrophoretic Force 

**Figure 1:**
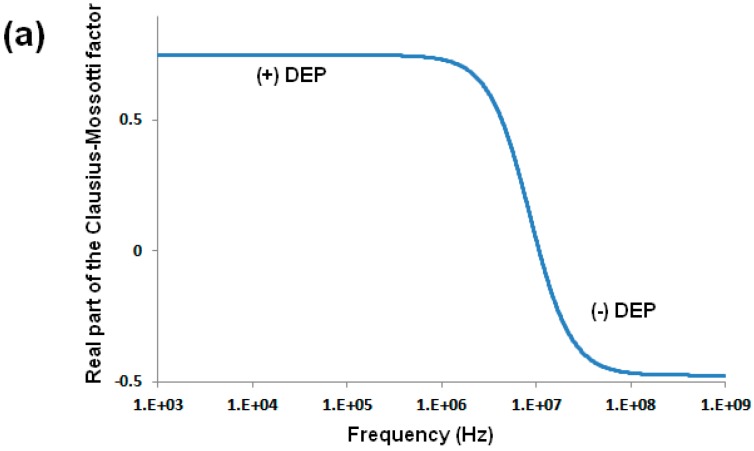

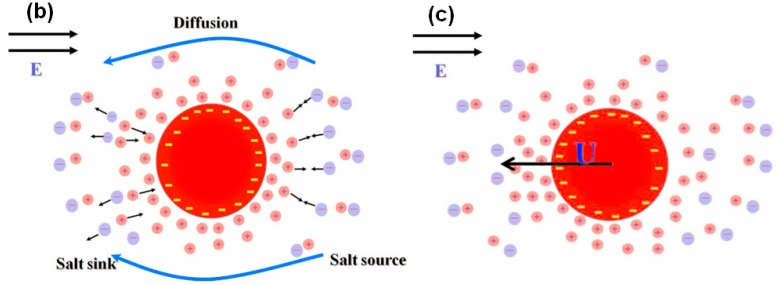
(**a**) Real part of the Clausius-Mossotti factor as a function of the electric field frequency for a solid spherical particle. For this plot, relative permittivities of 2.55 and 78.4 for the particle and the suspended medium, respectively, as well as conductivities of 10^−2^ and 10^−3^ S/m for the particle and medium, respectively, were considered. (**b**) Schematic representation of the polarization of the EDL for a negatively charged particle immersed in an electrolyte solution, with the formation ion concentration gradients. (**c**) Schematic representation of the EDL polarization caused by particle’s electrophoretic motion, the arrow coming from the particle represents the direction of the electrophoretic motion. In both illustrations the electric field direction is from left to right. Reprinted from [44], with the permission of AIP Publishing.

A dielectric particle immersed in dielectric medium will polarize under the presence of an electric field. The effective dipole moment for a spherical particle is given by:

(1) $$\vec{m}=4\pi \varepsilon_{m}r_{p}^{3}f_{CM}\vec{E}$$

where *ε_m_* is the medium permittivity, *r_p_* is the particle radius, and *f_CM_* is the Clausius-Mossotti factor (*f_CM_*). The dielectrophoretic force exerted on a spherical particle is derived from the dipole moment and is a function of particle size and its relative polarizability compared to that of the suspending medium:

(2) F→DEP=2πεmrp3Re(fCM)∇E2

where ∇*E*^2^ refers to the gradient of the electric field squared. Particle polarizability is expressed through the *f_CM_* which is estimated employing the complex permittivities of the particle and the suspending medium [40]:

(3) $$f_{CM}=\left(\frac{\varepsilon_{p}^{*}-\varepsilon_{m}^{*}}{\varepsilon_{p}^{*}+2\varepsilon_{m}^{*}}\right)$$

(4) $$\varepsilon_{}^{*}=\varepsilon -\left(j\sigma /\omega \right)$$

where *σ* and *ε* are real conductivity and permittivity values, respectively, *ω* is the angular frequency of the applied electric potential, and $j=\sqrt{-1}$. For spherical particles the magnitude of the *f_CM_* ranges from −0.5 to 1.0 [40]. The frequency of the applied electric potential is a critical parameter that determines the type of dielectrophoretic response of a particle from positive to negative DEP (Equations (3) and (4)). The cross-over frequency—defined as the frequency at which the DEP force and *f_CM_* are both zero—has been extensively used to characterize the dielectrophoretic behavior of submicron particles under AC electric potentials at frequencies above 10^5^ Hz [26,28,29,30]. Figure 1a illustrates the behavior of the real part of the *f_CM_* for a solid homogeneous spherical particle with properties similar to those of latex at electric field frequencies between 10^3^ and 10^9^ Hz. The image depicts how the particle behavior shifts from positive to negative DEP with a cross-over frequency between 10^6^ and 10^7^ Hz [26]. It has been reported that the magnitude of particle’s surface charge has significant effects on the cross-over frequency. Green and Morgan [25] separated nanoparticles by means of DEP based on surface charge differences by employing an array of electrodes and high frequency (~20 MHz) electric potentials. In their experiments, particles with a high surface charge had a positive dielectrophoretic response and particles with a low surface charge exhibited negative DEP. Similar results were reported in a later study [28] that was carried out with nanoparticles and high frequency (5 MHz) electric fields, where highly charged unmodified carboxylated particles experienced positive DEP and lowly charged antibody-coated particles displayed negative DEP. The aforementioned studies [25,28] clearly illustrate the effect of surface charge on particle dielectrophoretic behavior under conditions of high frequency electric fields. However, the dielectrophoretic behavior of particles at lower frequencies (<1000 Hz) is still an unexplored subject. The present study focuses on the dielectrophoretic behavior of particles employing DC and low frequency (<1000 Hz) electric potentials. Under these conditions the *f_CM_* can be simplified in terms of the real conductivities of the particle and the suspending medium [41]:

(5) $$f_{CM}=\frac{\sigma_{p}-\sigma_{m}}{\sigma_{p}+2\sigma_{m}}$$

The total particle conductivity (*σ_p_*) of polystyrene or latex particles can be described by the sum of the bulk conductivity (*σ_b_*) and the surface conductance (*K_S_*) [42]:

(6) $$\sigma_{p}=\sigma_{b}+2\frac{K_{S}}{r_{p}}$$

Typical values for *K_S_* are in the order of 1 nS [30]; although this value seems low, small changes in *K_S_* can generate significant changes in dielectrophoretic behavior of nanoparticles. Hughes et al. [30] demonstrated that particle surface conductivity is the sum of two surface conductance components. The first component accounts for charge movement in the Stern layer and the second component considers charge movement in the diffuse layer of the EDL. Therefore, particle total conductivity can be estimated as:

(7) $$\sigma_{p}=\sigma_{b}+2\frac{K_{Stern}}{r_{p}}+2\frac{K_{Diff}}{r_{p}}$$

where *K_Stern_* is the Stern layer conductance and *K_Diff_* is the diffuse layer conductance; a detailed derivation of these two conductance terms can be found elsewhere [30]. In the case of high conductivity suspending media—which does not apply to this study—interesting anomalous behavior has been reported at low frequency conditions where particles exhibit positive DEP [29]. 

For estimating particle total conductivity (*σ_p_*) the value of *σ_b_* can be considered negligible for latex and polystyrene [43]. Ermolina and Morgan determined the values of *K_Stern_* and *K_Diff_* for latex spheres with diameters of 44, 216, and 996 nm. Their findings illustrated that for smaller particles the magnitude of *K_Stern_* is only slightly lower than that of larger particles. However, the magnitude of *K_Diff_* for smaller particles is usually larger than that of larger particles (this effect increases with medium conductivity) [43]. These are two opposite effects that coupled together with the effect of particle radius (Equation (7)) result in smaller particles having a greater total conductivity than larger particles. 

### 2.2. Particle Polarization and Dipole Moment 

Particle polarization—the core of dielectrophoretic force—is the result of the interaction between the non-uniform electric field and the induced dipole moment on the particle. The dipole moment depends on the frequency of the electric field, particle surface charge and ratio of dielectric permittivities of the particle and suspending medium. Differences in the dipole moment can be exploited to separate or identify distinct types of particles [7].

In this study, the polarization and dielectrophoretic response of several types of charged polystyrene particles were assessed. When particles are immersed in an electrolyte solution, they develop an EDL. The ions contained in the EDL experience migration under the action of an electric field. This generates an EOF on the particle surface and produces an ionic current along the surface of the particle—resulting in the polarization of the EDL—which in turn alters the magnitude of the dipole moment. Figure 1b depicts a schematic representation of the EDL polarization for a negatively charged particle [44], where there is migration and convection of ions along the electric field direction ($\vec{E}$ is from left to right), and there is an exchange of ions between the diffuse layer and the electrolyte. Negative co-ions (blue color) migrate upstream while positive counter-ions (red color) migrate downstream, producing a depletion of ions upstream of the particle (salt sink) and an excess of ions downstream of the particle (salt source). This produces a phenomenon called “ion concentration polarization”, which is a gradient in ion concentration established along the length scale of the particle [7,44,45]. Furthermore, the charged particle will also exhibit an electrophoretic motion under the influence of the electric field; for a negatively charged particle the EP migration is towards the upstream direction. The electrophoretic motion enhances the polarization of the EDL, as depicted in Figure 1c, where an arrow coming from the particle is used to illustrate particle electrophoretic velocity [7,44,45]. 

The thickness of the EDL—relative to particle size—plays a major role in particle polarization. Several important theories have been developed to describe the interplay between the EDL thickness, the ion exchange between the EDL, and the bulk electrolyte and the dipole moment [7,44,45]. For our current operating conditions of low frequency (<1000 Hz) electric fields, the Dunkhin-Shilov (DS) theory is relevant. This theory, which is restricted to thin EDLs, assumes that the EDL is at quasi equilibrium with the suspending electrolyte, since the ions—due to low frequency condition—have enough time to reach local equilibrium. That is, the DS theory successfully predicts low frequency dispersion [22,44]. 

However, the effects of particle electrophoretic motion on the dipole moment are not fully considered by the DS theory, since it assumes that particles are stationary, which can only be justified when λ_D_ ≥ 0.1 (λ_D_ is Debye length normalized by the particle radius) [7,22,44]. For the current experimental conditions the values of λ_D_ for 100 nm and 200 nm diameter particles are 0.267 and 0.133, respectively, considering the 0.02 mM NaCl suspending medium employed. Therefore, for these particles, the electrophoretic contribution to the dipole moment has to be considered, since λ_D_ ≥ 0.1. In contrast, for the larger particles utilized in this study (500 nm and 1 μm in diameter), electrophoretic effects are not as significant since λ_D_ ≤ 0.1. This is in agreement with previous results by our group, where particles with diameter ≤200 nm exhibited positive dielectrophoretic behavior and particles with diameter ≥500 nm showed negative DEP [23]. However, in that previous report [23], all particles had a high surface charge. In the present study, some of the particles analyzed possess lower surface charge. The magnitude of the particle’s surface charge alters the particle zeta potential (ζ_p_) and the particle dipole moment. Zhao [44] analyzed the effect of ζ_p_ on the dipole moment coefficient; for these predictions a normalized zeta potential (ζ) was employed, which is defined as ζ_p_ normalized with the thermal voltage (~25 mV). Zhao reported that at low values of ζ the dipole moment coefficient shifts from negative to positive, almost in a linear fashion, and as ζ increases, it reaches a maxima at ζ ~ 4 (ζ_p_ ~ 100 mV). These estimations were valid for λ_D_ = 0.1 and λ_D_ = 0.3, which apply to our system [44]. 

Considering these findings, a particle with a lower surface charge would have a lower value for ζ_p_, which in turn would lead to a lower and perhaps negative value for the particle dipole moment coefficient, possibly producing a negative dielectrophoretic force on the particle. On the contrary, particles with higher surface charge would have a higher value of ζ_p_, resulting in a positive particle dipole moment coefficient, leading to a positive dielectrophoretic force on the particle. For a detailed discussion and derivation of the particle dipole moment, the reader is encouraged to review the report by Zhao [44]. 

In summary, the smaller particles in our study (100 nm and 200 nm in diameter) can exhibit positive or negative DEP depending on the magnitude of their surface charge. If the particle bears a sufficiently high surface charge (high ζ_p_), then electrophoretic motion can further enhance the particle dipole moment producing positive DEP effects on the particle. For a lowly charged particle (low ζ_p_), the particle’s electrophoretic motion is not sufficient to enhance the particle dipole moment, leading to a negative dipole moment and a negative dielectrophoretic particle motion [44]. Under our experimental conditions, larger particles (500 nm and 1 μm diameter) will always exhibit negative dielectrophoretic behavior due to their size. 

### 2.3. Particle Behavior in iDEP 

**Figure 2:**
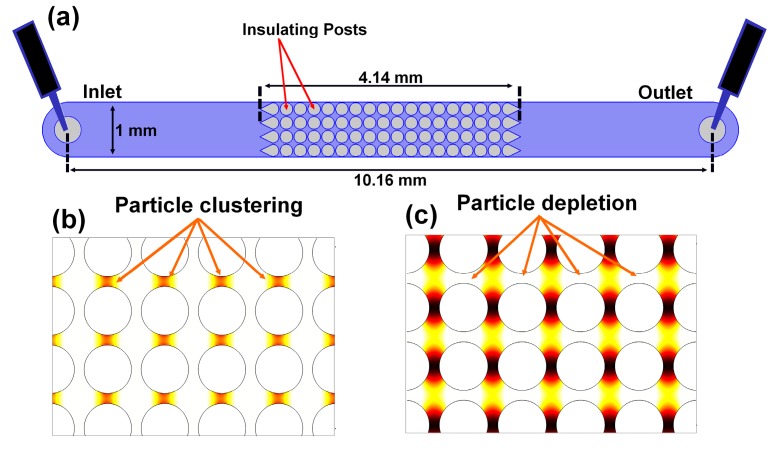
(**a**) Schematic representation of the iDEP microchannel used for experimentation, the image depicts the channel, the array of cylindrical insulating posts, the inlet and outlet reservoirs, as well as the location of the electrodes. (**b**) Modeling of particle velocity with COMSOL Multiphysics^®^ to illustrate positive DEP where particles cluster at the center of the constriction between the cylindrical posts. (**c**) Modeling of negative DEP where particles are repelled from the constriction region, creating a region depleted of particles at the constriction between the cylindrical posts.

In iDEP systems, particles are exposed to linear EK (superposition of EP and EOF) and DEP forces. Many iDEP systems usually consist of a microchannel with an embedded array of insulating structures, such as the one illustrated in Figure 2a. Our group has studied particle polarization in these types of devices employing a mathematical model built with COMSOL Multiphysics^®^ and extensive experimentation [23]. Since particles are affected by linear EK and DEP, particle velocity, which accounts for both forces, is an ideal parameter for predicting the regions where particles will gather under the influence of an electric field in an iDEP system. Considering a microchannel with cylindrical insulating structures, positive DEP would lead to particle clustering at the center of the constriction between the posts, where the particle velocity is the highest—since particles are attracted to these regions, as depicted in Figure 2b. In contrast, negative DEP would produce a region of particle depletion at the constriction between the posts, since the particles are being repelled from the regions with the higher particle velocity, as illustrated in Figure 2c. Details about the COMSOL model are published elsewhere [46]. These particular simulations are for 100 nm particle under the effect of 3000 V applied across the length of the microchannel illustrated in Figure 1a. For the positive dielectrophoretic behavior image, a combined zeta potential *ζ_c_* (defined as *ζ*_c_ = *ζ_p_* + *ζ_w_*) of −15 mV was assumed, considering a particle with high negative charge, where *ζ_w_* represents the wall zeta potential. For the negative dielectrophoretic behavior image, it was assumed that the particle has a low charge depicted by a *ζ_c_* = −75 mV. The use of the zeta potentials ensured that a flat velocity profile was considered for EOF in the COMSOL simulations.

## 3. Materials and Methods 

### 3.1. Microfluidic Devices 

Experiments were conducted with microchannels made from Polydimethylsiloxane (PDMS, Dow Corning, Midland, MI, USA) using standard soft lithography techniques [47]. A mold for the microchannels was created using a silicon wafer (Silicon Inc., Boise, ID, USA) and SU-8 3050 photoresist (MicroChem, Newton, MA, USA). PDMS was then casted onto the SU-8 negative replica and once cured, sealed onto a 10-cm PDMS coated glass wafer using a plasma corona wand (Electro Technic Products, Chicago, IL, USA) to activate both PDMS surfaces. The microchannels employed consisted of 10.16 mm long channels, 1 mm wide, 40 μm deep and contained one inlet and one outlet liquid reservoirs. The cylindrical posts employed were 200 μm in diameter and arranged 220 μm center to center. The spacing between the posts and the channel wall was 10 μm. The array of cylindrical insulating structures consisted of 72 posts located in the middle of the channel, arranged as 18 rows of four posts each (Figure 2a). The first column of posts featured a “dove tail” geometry which was added to prevent clogging in the channel by having particles splattered on the first column of posts. 

### 3.2. Particles and Suspending Media 

Fluorescent polystyrene microspheres of sizes ranging from 0.1 to 1.0 μm obtained from two different providers—Invitrogen (Eugene, OR, USA) and Magsphere (Pasadena, CA, USA)—were employed. The microspheres ranged in surface charge magnitude, fluorescence color, type of surface functionalization and concentrations employed. Specific details of the microparticles used are listed in Table 1. 

The suspending medium used in experimentation consisted of diluted salt solutions of K_2_HPO_4_ or NaCl. The pH and conductivity were adjusted by the addition of a 0.1 N KOH solution to a pH ~ 7.5 and a conductivity in the range of 35–100 μS/cm. Tween 20 surfactant was also added to the solution at a concentration of 0.05% vol/vol in order to minimize aggregation of particles. Specific information about the buffer solution used in each experiment is included in the figure legends in the results section. In order to ensure stable electroosmotic flow and avoid adhesion of particles to the surface of the channel, devices were soaked in buffer two hours prior to experimentation. 

### 3.3. Equipment and Software 

All experiments were imaged using a Leica DMi8 inverted microscope (Leica Microsystems Wetzlar, Wetzlar, Germany) and a Leica DFC7000 T video camera. The microscope was equipped with two fluorescence filter sets: a GFP filter set and a Texas Red (TXR) filter set. Two distinct voltage supplies were employed to apply AC or DC electric potentials. A waveform generator (Agilent 33500 Series, Santa Clara, CA, USA) and amplifier (Model: PZD700A2, Trek Inc., Lockport, NY, USA) were used to apply AC electric potentials up to 3600 V peak-to-peak (Vpp). DC potentials up to 6000 V and low frequency (<1000 Hz) AC potentials were applied with a voltage sequencer (Model HVS6000D; LabSmith, Livermore, CA, USA). 

### 3.4. Experimental Procedure 

Experiments were conducted using a clean microchannel filled with the selected suspending medium. The channels were reversibly sealed to a vacuum chuck manifold (LabSmith, Livermore, CA, USA) employing a vacuum pump (Model 400–3910; Barnant Company, Barrington, IL, USA). Platinum wire electrodes were placed at the reservoirs and an electric potential was applied to generate a dielectrophoretic response in the particles. 

For all experiments, a sample of 10 μL of the particle suspension was employed (Table 1). Particle response was observed and recorded for every trial using the inverted microscope and video camera. The microchannels were re-conditioned in between uses by soaking them in 0.1 N KOH for 2 h and then soaked in DI water for 1 h in order to ensure a negative surface charge on the PDMS surface and a stable electroosmotic flow. To avoid any undesired effects (e.g., electrolysis, Joule heating, etc.) due to the high applied voltages, all experiments were limited to 30–60 s. During this time window no negative effects were observed.

## 4. Results and Discussion 

### 4.1. Effect of Particle Size on Polarization 

The effect of size in dielectrophoretic trapping has been explored by our group before [23]. It was shown that under DC and low frequency AC potentials sub-micron particles could exhibit positive or negative dielectrophoretic behavior. Highly charged polystyrene particles with diameters <200 nm exhibited positive DEP, while particles with diameters >500 nm exhibited negative DEP [23], a particle size threshold for the shift from positive to negative DEP was observed to be between 200 nm and 500 nm diameter

**Figure 3:**
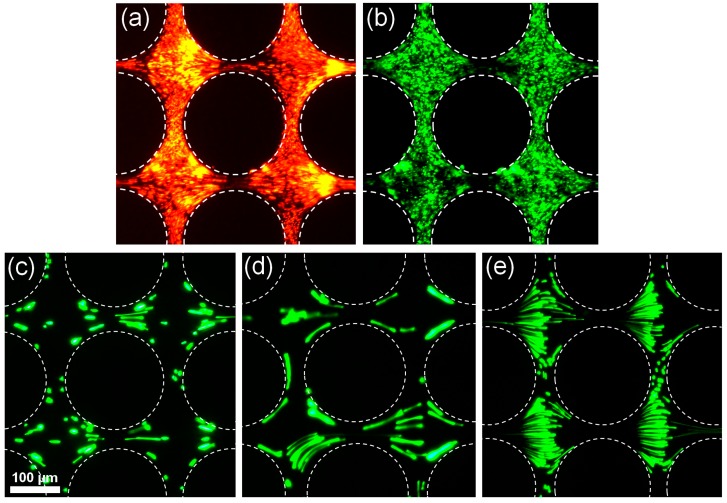
Negative dielectrophoretic response of 500 nm and 1 μm particles under AC potentials—all images show particle depletion at the constrictions between the cylindrical posts. All of these particles are from Invitrogen. (**a**) 500 nm green aminated particles at 2800 Vpp and 70 Hz. (**b**) 500 nm red carboxylated particles at 2800 Vpp and 70 Hz. (**c**) 1 μm green carboxylated particles 2800 Vpp and 1000 Hz. (**d**) 1 μm green aminated particles at 2800 Vpp and 1000 Hz. (**e**) 1 μm green sulfated particles at 2000 Vpp and 1000 Hz. The suspending mediums employed were 0.02 mM NaCl with pH = 7.5 and conductivity = 35 μS/cm for the 500 nm particles (images **a**–**b**); and 0.4 mM K_2_HPO_4_ with pH =7.5 and conductivity = 100 μS/cm for the 1 μm particles (images **c**–**e**).

In order to assess the effect of the magnitude of the particle’s surface charge and type of surface functionalization on dielectrophoretic behavior, experiments with particles over the size threshold of 500 nm in diameter were conducted. Due to their size, these particles are expected to depict negative DEP. Particles with amine, carboxyl, and sulfate surface functionalizations, with diameters of 500 nm and 1 μm, and surface charges ranging from 0.01 to 0.18 meq/g (Table 1) were exposed to low frequency AC potentials. As illustrated in Figure 3, all five distinct particle types employed in this set of experiments exhibited negative dielectrophoretic motion. A clear depletion of particles is observed in the constriction between the insulating posts; where the highest electric field gradient is present. Figure 3a,b show the behavior for 500-nm particles, and Figure 3c–e display the behavior for 1 μm particles. It is important to note that a 0.4 mM K_2_HPO_4_ buffer with a higher ionic strength was used for the experiments conducted with 1 μm particles, to strengthen the negative dielectrophoretic behavior; in particular the behavior shown in Figure 3e is a very well-defined depiction of negative DEP. From these findings, it is demonstrated that due to their large size these particles will exhibit negative dielectrophoretic motion regardless of particle functionalization (amine, carboxyl, or sulfate) or magnitude of surface charge. These results are in agreement with a previous publication from our group [17]. The next section reports the experiments conducted with submicron particles (diameter < 500 nm), where two distinct types of dielectrophoretic behaviors were observed. 

### 4.2. Effect of Particle Charge Magnitude for Smaller Particles (dp < 500 nm)

**Figure 4:**
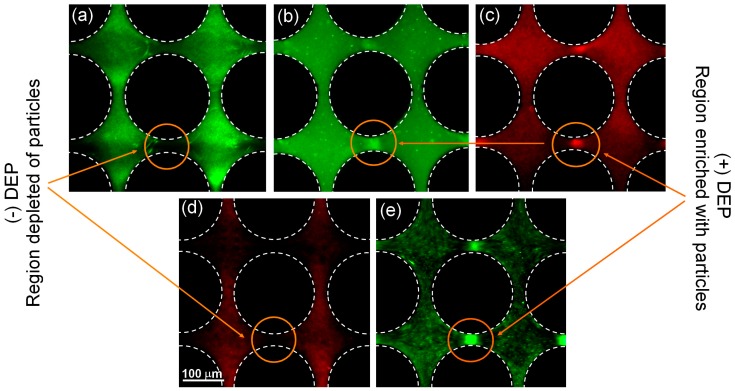
Dielectrophoretic response of 100 nm and 200 nm particles under AC potentials. Images a and d correspond to lowly charged particles and illustrate negative dielectrophoretic response with a depletion of particles at the constriction between the posts. Images b, c, and e correspond to highly charged particles and illustrate positive dielectrophoretic response, where particles form a cluster at the constriction between the posts. (**a**) Negative DEP—100 nm green aminated particles from Magsphere at 2800 Vpp and 1000 Hz. (**b**) Positive DEP—100 nm green carboxylated particles from Magsphere at 2800 Vpp and 1000 Hz. (**c**) Positive DEP—100 nm red carboxylated particles from Invitrogen at 2800 Vpp and 1000 Hz. (**d**) Negative DEP—200 nm red aminated particles from Magsphere at 3600 Vpp and 200 Hz. (**e**) Positive DEP—200 nm green carboxylated particles from Invitrogen at 3000 Vpp and 200 Hz. All experiments were carried out with 0.02 mM NaCl with pH = 7.5 and conductivity = 35 μS/cm as suspending medium.

The effect of surface charge on the dielectrophoretic motion of particles with diameters <500 nm was studied experimentally as a function of surface charge magnitude and type of functionalization (amine and carboxyl). Microspheres obtained from two different providers, Invitrogen and Magsphere (Table 1) were employed, where Invitrogen particles were observed to have higher surface charge magnitudes than Magsphere particles by an order of magnitude, as shown in the Supplementary Information (Table S1). Submicron particles, 100 nm and 200 nm diameter in size, from both providers were observed to exhibit distinct types of dielectrophoretic behavior under low frequency AC signals. It was assumed that particles provided by Magsphere had low surface charge, while particles provided by Invitrogen carried higher surface charges, as defined by data provided by the manufacturer (Table 1). The results from these experiments are included in Figure 4. It was observed that particles with a low surface charge magnitude (Figure 4a,d) exhibit negative dielectrophoretic behavior—as depicted by particle depletion at the constriction between the posts. This is in agreement with the discussion presented in Section 2.2, where it was explained that low charge leads to a low ζ_p_ for the particle, which in turn decreases the electrophoretic contribution to the dipole moment. Although the exact charge magnitude is unknown, these aminated particles are expected to have lower surface charge magnitude than carboxylated particles. Microsphere manufacturers have very robust processes and experience producing highly charged carboxylated particles, while usually lower charge densities are achieved for other functional groups such as amine and sulfate. Meanwhile, carboxylated particles which possess a higher surface charge magnitude (Figure 4b,c,e) presented positive dielectrophoretic particle capture, depicted by the formation of a cluster of particles at the center of the constrictions between two posts. These findings are in agreement with the discussion included in Section 2.2. Highly charged particles possess a higher ζ_p_, which leads to enhancement of the particle dipole moment due to particle electrophoretic motion. These results illustrate that the magnitude of the surface charge is the main parameter dictating the dielectrophoretic response of the particles in this size range, while the type of the surface functionalization does not seem to have a significant effect. That is, particles below the size threshold of 500 nm could exhibit positive or negative dielectrophoretic behavior depending on the magnitude of their surface charge, while larger particles (Figure 3) would exhibit only negative dielectrophoretic behavior dictated by their size, rather than the magnitude of the surface charge present on the particle. 

### 4.3. Application: Dielectrophoretic Differentiation of Sub-Micron Particles by Exploiting Charge Differences 

The previous experiments demonstrated that particles with different surface charge will exhibit distinct dielectrophoretic behaviors. To further assess the potential of exploiting charge difference by means of iDEP, a set of DC-iDEP experiments were conducted using a mixture of two types of particles of the same size, same shape, and same substrate material, but different surface charge magnitude (Table 1). The mixture contained 100 nm-red-carboxylated particles by Invitrogen and 100 nm-green-aminated particles by Magsphere. The red-carboxyl particles in the mixture are considered to have a higher surface charge than the green-amine particles, as depicted by the results in Figure 4a,c under AC electric fields. 

**Figure 5:**
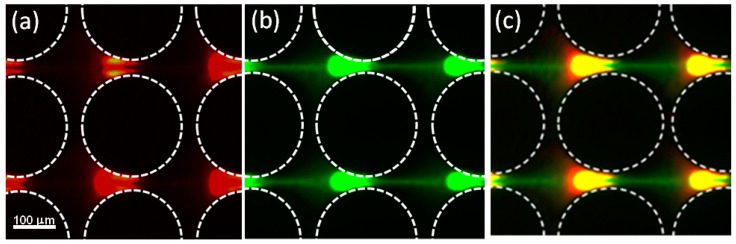
Dielectrophoretic response of a mixture of two types of 100 nm particles under a DC electric potential of 1300 V. The particle mixture is composed of 100 nm red carboxylated particles (high charge) from Invitrogen and 100 nm green aminated particles from Magsphere (low charge). (**a**) Image showing only the red polystyrene particles (obtained with the TXR filter) exhibiting positive DEP. (**b**) Image showing only the green aminated particles (obtained with the GFP filter) exhibiting negative DEP. (**c**) Image depicting both particle types, obtained by stacking the two previous images. This experiment was carried out with 0.02 mM NaCl with pH = 7.5 and conductivity = 35 μS/cm as suspending medium.

Figure 5 illustrates the behavior of the mixture of two types of 100 nm particles exposed to 1300 V DC and observed under two different fluorescence filter sets (GFP filter and TXR filter). The use of two different filters allowed for visualization of each individual type of particle within the mixture. Figure 5a shows the mixture under the TXR filter to focus only on the red particles, while Figure 5b was taken with the GFP filter to focus only on the green particles. Further analysis of these particle images suggests that the red-carboxyl particles—which bear a higher surface charge—exhibit positive DEP; as these particles are fully trapped at the applied potential—these are no particles escaping the dielectrophoretic trap. This illustrates that DEP is able to overcome EK under these conditions, meaning that electrophoretic motion (which is opposite to EOF) is sufficient to decrease the overall magnitude of EK towards the outlet. In contrast, Figure 5b shows the behavior of lowly charged particles, where DEP is not able to overcome EK completely (EP does not sufficiently decrease EK), and particles are escaping the dielectrophoretic traps—as evidenced by the green fluorescent particle stream that comes out of each particle cluster. It is important to note that the clusters of green particles feature a “round” back, this means that these particles are more affected by EK motion than the red ones. As previously reported by our group [46], clusters of particles that exhibit negative DEP have a “back” (upstream direction) that is defined by EK, which results in a round shape. The clusters of green particles also feature a flatter “front” than the red particles, which further supports that the green particles are exhibiting negative DEP [46]. Figure 5c presents the stacking of the images in Figure 5a,b, where the leakage of green particles is evident. 

**Figure 6:**
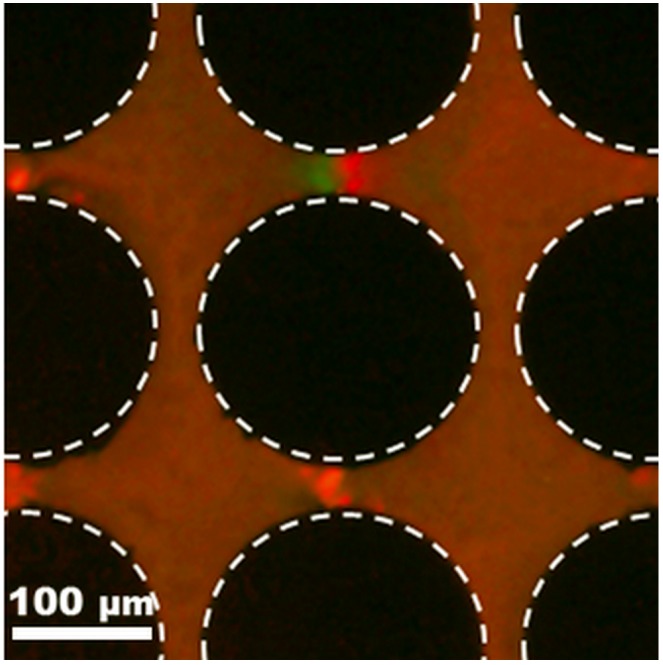
Dielectrophoretic response of a mixture of two types of 100 nm particles under an applied AC electric potential of 3200 Vpp at 1000 Hz. The particle mixture is composed of 100 nm red carboxylated particles (high charge—positive DEP) from Invitrogen and 100 nm green aminated particles from Magsphere (low charge—negative DEP). The red particles exhibit a clear positive DEP behavior and are clustered at the center of each constriction, while the green particles depict a weaker response of negative DEP (slight repulsion from the constriction region). This experiment was carried out with 0.02 mM NaCl with pH = 7.5 and conductivity = 35 μS/cm as suspending medium.

This mixture of two distinct types of 100 nm particles (low charge vs. high charge) was also analyzed under AC potentials; these results are included in Figure 6. It can be observed from the figure that the red particles (positive DEP) are trapped at the center of the constriction between two posts. Conversely, the green particles (negative DEP) exhibit a weaker dielectrophoretic response and are not clustered at the center of the constriction; instead they are gathered to the left of the red particles at one of the constrictions. Furthermore, no appreciable dielectrophoretic response from the green particles is found at the other constrictions between the posts. Since negative DEP is particle repulsion—not particle trapping—it is more challenging to observe, as highlighted by Green and Morgan [25] in their work with similar subpopulations of particles of the same size (93 nm diameter). Our 100 nm particles are small, which makes it difficult to generate sufficient dielectrophoretic force to produce discernible particle movement and create regions of particle depletion in our current experimental set-up. 

The results from this experiment with a particle mixture demonstrate how submicron particles that are the same size, same shape, and made from the same substrate material can have different dielectrophoretic behaviors as a result of distinct surface charge magnitudes. These findings reveal the potential in exploiting differences in surface charge magnitude to separate very similar submicron particles based on their polarization behavior and dielectrophoretic response by employing simple iDEP microdevices under DC and low frequency (<1000 Hz) AC potentials. 

## Figures and Tables

**Table 1:** Fluorescent polystyrene microspheres information including diameter (μm), fluorescence (Ex/Em), provider, charge (meq/g), type of surface functionalization and particle concentration employed.

Diameter (μm)	Fluorescence (Ex/Em)	Provider	Charge (meq/g)	Surface Functionalization	Concentration (Particles/mL)
0.1	Green (480/501)	Magsphere	Not Available	Amine	9.09 × 10^11^
0.1	Green (480/501)	Magsphere	0.0680	Carboxyl	9.09 × 10^11^
0.1	Red (580/605)	Invitrogen	0.3850	Carboxyl	9.09 × 10^11^
0.2	Green (505/515)	Invitrogen	0.5810	Carboxyl	5.68 × 10^10^
0.2	Red (538/584)	Magsphere	Not Available	Amine	5.68 × 10^10^
0.5	Green (480/501)	Magsphere	Not Available	Amine	1.46 × 10^9^
0.5	Red (580/605)	Invitrogen	0.3160	Carboxyl	1.46 × 10^9^
1.0	Green (505/515)	Invitrogen	0.0227	Amine	2.18 × 10^8^
1.0	Green (505/515)	Invitrogen	0.1826	Carboxyl	2.18 × 10^8^
1.0	Green (505/515)	Invitrogen	0.0170	Sulfate	2.18 × 10^8^

## Supplementary Materials

The following are available online at www.mdpi.com/2072-666X/8/8/239/s1, Table S1: Fluorescent polystyrene microspheres information including diameter (μm), provider, charge (meq/g) and type of surface functionalization.
