# Supplementary material for: Assessment of Sub-Micron Particles by Exploiting Charge Differences with Dielectrophoresis
Source: Micromachines (Basel). 2017 Aug 2;8(8):239. doi: 10.3390/mi8080239 (PMC6190034; doi:10.3390/mi8080239)
Supplement: Supplementary file 1 [file micromachines-08-00239-s001.pdf]

Micromachines **2017**, *8*, 239; doi:10.3390/mi8080239

Assessment of Sub-Micron Particles by Exploiting Charge Differences with Dielectrophoresis

Maria F. Romero-Creel, Eric Goodrich, Danielle V. Polniak and Blanca H. Lapizco-Encinas *

Table S1. Comparison of particle surface charge magnitude for particles from two providers: Invitrogen and Magsphere.

Diameter (μm)	Provider	Charge (meq/g)	Surface Functionalization
0.1	Magsphere	0.0680	Carboxyl
0.1	Invitrogen	0.2839	Carboxyl
0.29	Magsphere	0.0360	Carboxyl
0.2	Invitrogen	0.5180	Carboxyl
0.5	Magsphere	0.0260	Carboxyl
0.5	Invitrogen	0.3156	Carboxyl
1.0	Magsphere	0.0220	Carboxyl
1.0	Invitrogen	0.1826	Carboxyl
